# Early prediction of long-term upper limb spasticity after stroke: Part of the SALGOT study

**DOI:** 10.1212/WNL.0000000000001908

**Published:** 2015-09-08

**Authors:** Arve Opheim, Anna Danielsson, Margit Alt Murphy, Hanna C. Persson, Katharina Stibrant Sunnerhagen

**Affiliations:** From the Institute of Neuroscience and Physiology (A.O., A.D., M.A.M., H.C.P., K.S.S.), Rehabilitation Medicine, Sahlgrenska Academy, University of Gothenburg, Sweden; and Sunnaas Rehabilitation Hospital (A.O.), Nesoddtangen, Norway.

## Abstract

**Objective:**

To identify predictors and the optimal time point for the early prediction of the presence and severity of spasticity in the upper limb 12 months poststroke.

**Methods:**

In total, 117 patients in the Gothenburg area who had experienced a stroke for the first time and with documented arm paresis day 3 poststroke were consecutively included. Assessments were made at admission and at 3 and 10 days, 4 weeks, and 12 months poststroke. Upper limb spasticity in elbow flexion/extension and wrist flexion/extension was assessed with the modified Ashworth Scale (MAS). Any spasticity was regarded as MAS ≥1, and severe spasticity was regarded as MAS ≥2 in any of the muscles. Sensorimotor function, sensation, pain, and joint range of motion in the upper limb were assessed with the Fugl-Meyer assessment scale, and, together with demographic and diagnostic information, were included in both univariate and multivariate logistic regression analysis models. Seventy-six patients were included in the logistic regression analysis.

**Results:**

Sensorimotor function was the most important predictor both for any and severe spasticity 12 months poststroke. In addition, spasticity 4 weeks poststroke was a significant predictor for severe spasticity. The best prediction model for any spasticity was observed 10 days poststroke (85% sensitivity, 90% specificity). The best prediction model for severe spasticity was observed 4 weeks poststroke (91% sensitivity, 92% specificity).

**Conclusions:**

Reduced sensorimotor function was the most important predictor both for any and severe spasticity, and spasticity could be predicted with high sensitivity and specificity 10 days poststroke.

Upper limb spasticity has been found to be associated with reduced arm function and low levels of independence, and with a 4-fold increase in direct care costs during the first year poststroke.^1–5^ The prevalence of upper limb spasticity in all patients 12 months poststroke varies from 17% to 38%^6–10^ and was found to be 46% in patients with initial impaired arm function.^5^ It has been found that 4%–13% of patients need treatment for spasticity 6–12 months poststroke.^6,9^ Previous studies during the first 10 days poststroke have identified several predictors for spasticity 3–12 months poststroke, e.g., reduced sensorimotor function and activities of daily living (ADL), muscle weakness, left-sided paresis, and smoking.^8,9,11,12^ These studies were relatively small (n = 47),^9^ assessed patients several days after stroke onset,^11^ or assessed spasticity in both upper and lower limbs. Whether early assessments of upper limb function and impairments can predict the occurrence and degree of upper limb spasticity 12 months poststroke with good accuracy is uncertain. The optimal time for early prediction of upper limb spasticity 12 months poststroke is also unknown.^12^ This information is of clinical relevance, as patients with an increased risk of developing spasticity-related impairments, complications, and increased disability may be identified.^1,13^ The study aims were to identify predictor variables and the optimal time for early prediction of any spasticity and severe spasticity in the upper limb 1 year poststroke.

## METHODS

All patients with first-ever stroke in an 18-month period in 2009–2010 who were admitted to the largest of 3 acute stroke units at the Sahlgrenska University Hospital, Gothenburg, Sweden, within 3 days after stroke onset were eligible for consecutive screening for inclusion in the present study, which was a part of the Stroke Arm Longitudinal Study at the University of Gothenburg (SALGOT). In the SALGOT study, the recovery of upper extremity function was investigated in a nonselected sample during the first year poststroke.^14^ All included patients had ischemic or hemorrhagic stroke^15^ for the first time, were over 18 years old, and had impaired upper extremity function, which was assessed at day 3 with the Action Research Arm Test (ARAT) (0–57)^16^ and defined as <57 points. The study sample size estimation (n = 88) for SALGOT was to determine a medium change of 6 points (10%) on ARAT, with a power of 0.8 and a significance level of 0.05. With an expected dropout rate of 30%, the aim was to include 120 patients.^14^

### Standard protocol approvals, registrations, and patient consents.

Study approval was provided by the Regional Ethics Committee of the Western region of Sweden (Registration number 225/08), and written informed consent was obtained. The study is registered at www.clinicaltrials.gov (NCT 01115348).

### Assessment procedure.

In SALGOT, the patients were assessed 9 times during the first year: at admission; at 3 and 10 days; at 3, 4, and 6 weeks; and at 3, 6 and 12 months poststroke. In the current study, data from admission, 3 and 10 days, 4 weeks, and 12 months were used. Predominantly, the assessments were carried out by 3 physiotherapists and were performed according to a standardized protocol.^14^ A majority of the assessments were performed at the university hospital. If traveling was not possible for the patient, the assessments were conducted in the patient's home, nursing home, or rehabilitation unit.

### Variables.

#### Predictor variables collected at admission (day 0).

Clinical characteristics and assessments routinely registered at admission during the acute stage of stroke were selected as potential predictor variables (age, sex, ischemic or hemorrhagic stroke, side of stroke, and smoking in the last 3 months). Stroke localization was classified using the Oxfordshire Classification^17^ and ischemic stroke was classified after cause of lesion using the Trial of Org 10172 in Acute Stroke Treatment criteria.^18^ The initial severity of stroke and arm paresis was assessed with the 0- to 42-point ordinal NIH Stroke Scale (NIHSS) and the NIHSS arm subscale (0–4), respectively.^19^ NIHSS arm was treated as a categorical variable with 0 as the reference category.

#### Predictor variables collected at 3 and 10 days and 4 weeks poststroke.

Common clinical assessment scales of sensorimotor impairments assessed at 3 and 10 days and 4 weeks poststroke were selected as potential predictors. Sensorimotor function in the upper limb was assessed with the motor function part (sections A–D) of the Fugl-Meyer Assessment Upper Extremity Scale (FMA-UE).^20^ The FMA-UE (sections A–D) includes 33 active motor function tests, where a higher score indicates a better performance (0–66). The nonmotor domains of the same scale (sections H–J) were used to assess sensation (0–12), joint pain (0–24), and range of motion (ROM) during passive joint motions (0–24); lower scores indicate reduced sensation, more pain, and reduced ROM, respectively. Spasticity in elbow flexors, elbow extensors, wrist flexors, and wrist extensors were assessed with the 6-level modified Ashworth Scale (MAS).^21^ The original MAS categories were reordered into integers between 0 and 5, to incorporate the score 1+. MAS were dichotomized, and spasticity was considered to be present if the MAS score was ≥1 in any of these muscle groups.

#### Dependent variables.

At 12 months poststroke, spasticity was reassessed in a similar way as previously. Any spasticity was considered if the MAS score was ≥1, and severe spasticity was considered if the MAS score was ≥2 in any of the muscle groups.^22^

### Statistical methods.

**Figure 1:**
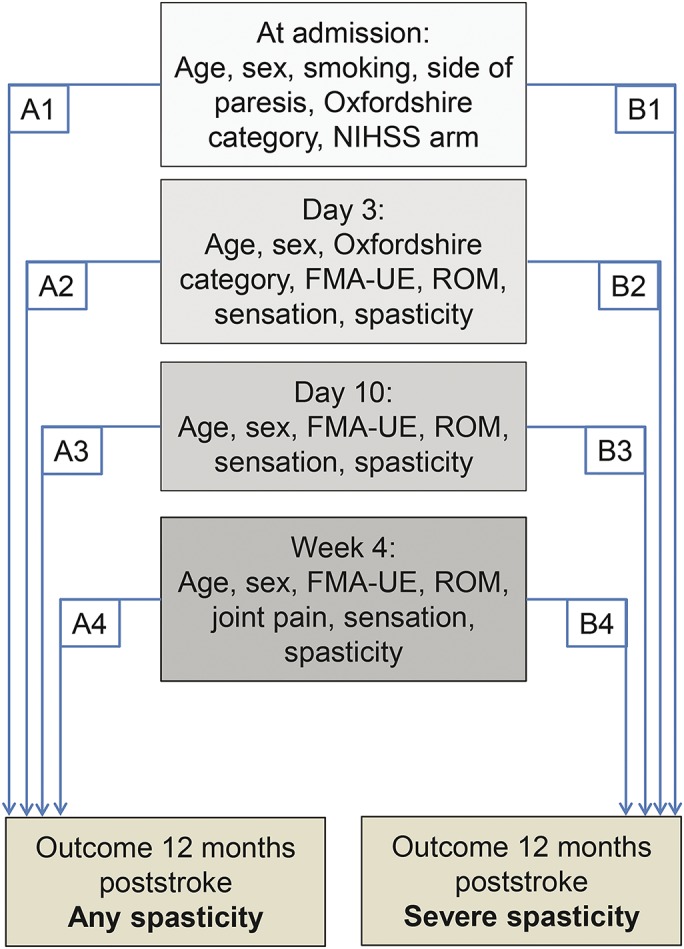
The multivariate logistic regression analyses models At day 3, Oxfordshire category and spasticity was included only in model B2. FMA-UE = Fugl-Meyer Assessment Upper Extremity scale; NIHSS = NIH Stroke Scale; ROM = range of motion.

Continuous and normally distributed variables are presented with means and SDs. Ordinal and non-normally distributed variables are presented with medians and 1st and 3rd quartile (Q1–Q3). Univariate logistic regression analyses were used to assess the relationship between the potential predictor variables and the outcome variable. The predictor variables were tested for correlations, and when 2 variables had a high correlation (*r* > 0.8), one was omitted before multivariate logistic regression analysis. The multivariate logistic regression analyses were used to predict the presence of (A) any spasticity and (B) severe spasticity at 12 months poststroke. In both (A) and (B), 4 models (A1–A4 and B1–B4) were found and compared (figure 1). The criteria for including a potential predictor variable in the multivariate logistic regression analyses were as follows: (1) significant predictor identified in previous studies, with the condition that if these variables were not found predictive in models A/B1, they were not included in models A/B2–4, and (2) univariate logistic regression *p* value <0.20. Age and sex were included in all models. The variables in the multivariate logistic regression analysis are shown in figure 1.

In the multivariate logistic regression analysis, the enter method was used stepwise and nonsignificant variables were removed manually one at the time, to ensure that only significant variables (*p* < 0.05) were included in the final model. To control for possible nonlinearity between summed ordinal predictors and the dependent variable, a multivariate model with the squared predictor variables (FMA-UE^2^ and Sensation^2^) was tested. Nonsignificant associations were interpreted as nonlinearity not shown.^23^ The results are presented with unstandardized coefficients, *p* values, and odds ratios with 95% confidence intervals (95% CIs).^24^ The sensitivity (%), specificity (%), positive likelihood ratio (PLR), negative likelihood ratio (NLR), and the corresponding 95% CI for each of the models was calculated using MedCalc for Windows, version 12.7.7.0 (MedCalc Software, Ostend, Belgium). All other statistical calculations were performed using IBM SPSS statistics 21.0 (IBM, Armonk, NY). The Strengthening the Reporting of Observational Studies in Epidemiology (STROBE) guidelines were followed.^25,26^

## RESULTS

### Participants.

In total, 763 patients were admitted to the stroke unit during the inclusion period, 117 of whom were included in the study and assessed at day 3 (SD 1). There were no significant differences between the participating and nonparticipating patients in terms of sex or NIHSS score. The nonparticipating patients were significantly older (76.0 [SD 13.1] vs 69.2 [SD 13.2] years, *p* < 0.001), and fewer of them had hemorrhagic infarction (6% vs 16% [χ^2^ = 4.92, *p* = 0.027]).

In total, 76 patients were assessed 12 months poststroke (35% dropout) and included in the logistic regression analysis. The 2 main causes for dropout were death (n = 14) and study withdrawal (n = 7). The clinical characteristics of the 76 patients included in the logistic regression analysis are shown in table 1.

**Table 1:**
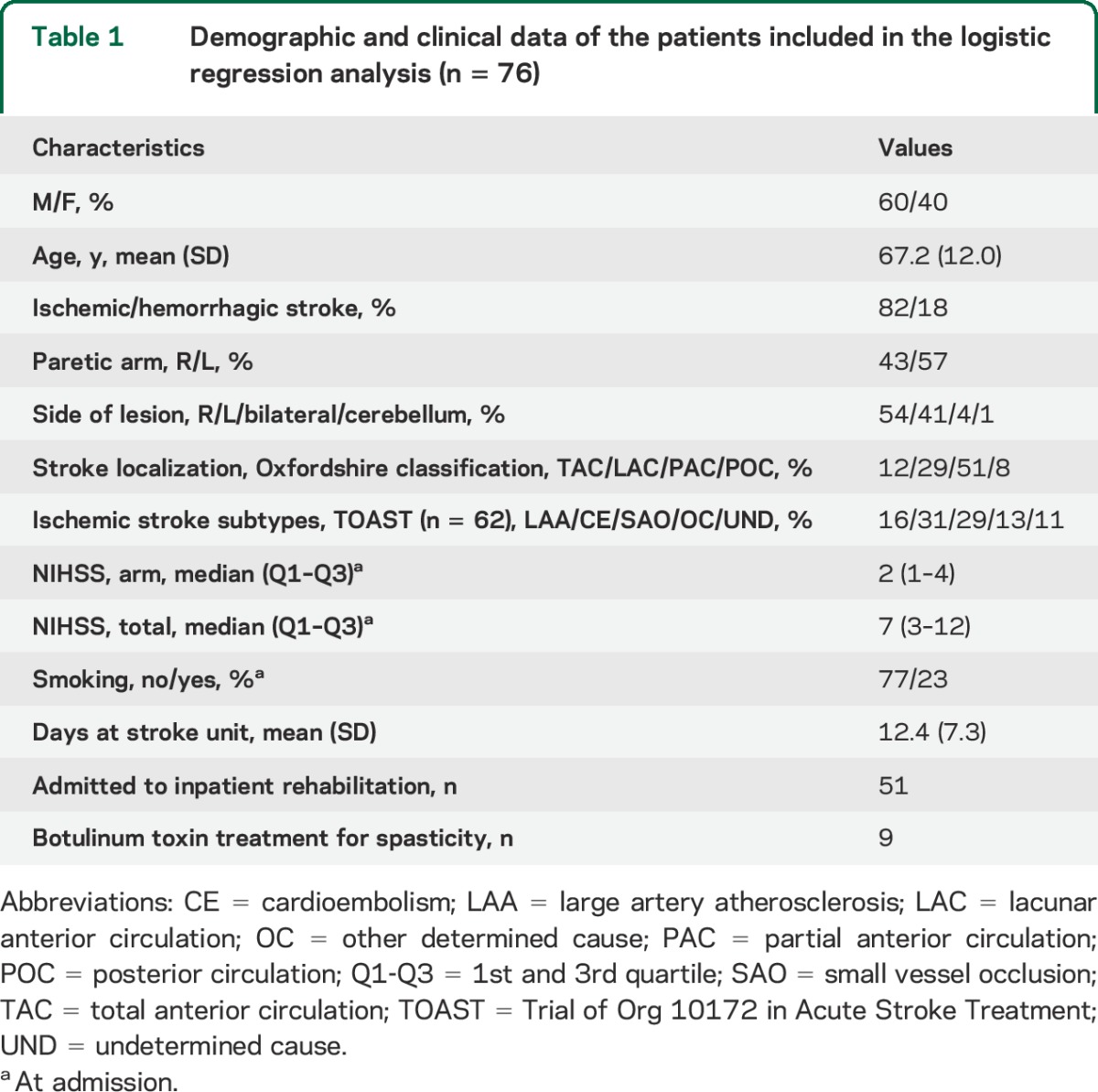
Demographic and clinical data of the patients included in the logistic regression analysis (n = 76)

At 3 and 10 days and 4 weeks, 24%, 43%, and 46% of the 76 patients were assessed with any spasticity, respectively. At 12 months, 46% and 29% were assessed as having any spasticity and severe spasticity, respectively.

### Prediction of any spasticity 12 months poststroke.

**Figure 2:**
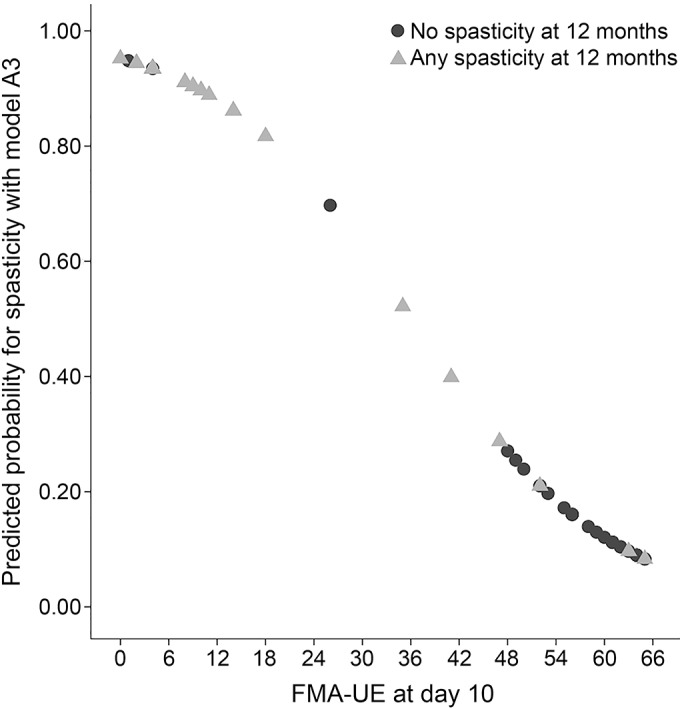
Predicted probabilities for spasticity 12 months poststroke and Fugl-Meyer Assessment Upper Extremity scale at day 10 The figure shows the predicted probabilities for any spasticity at 12 months poststroke as found in model A3 (10 days poststroke), in relation to sensorimotor function at day 10, assessed with the Fugl-Meyer Assessment Upper Extremity scale (FMA-UE).

The univariate logistic regression analysis for all potential predictor variables for any spasticity and severe spasticity is presented in tables e-1 and e-2 on the *Neurology*® Web site at Neurology.org. The multivariate logistic regression analysis results are presented in table 2. In models A2 and A3, the FMA-UE was a significant predictor. In model A4, age at stroke onset was a significant predictor in addition to FMA-UE, and higher FMA-UE scores and higher age were associated with reduced probability for spasticity. The sensitivity, specificity, PLR, and NLR of model A3 indicated that this model had the highest predictive value (table 3). The fit of prediction model A3 was assessed in a scatterplot of the predicted probabilities in relation to FMA-UE at day 10 (figure 2). The squared FMA-UE variable (FMA-UE^2^) was not significant in models A2–A4, and nonlinearity could not be shown.

**Table 2:**
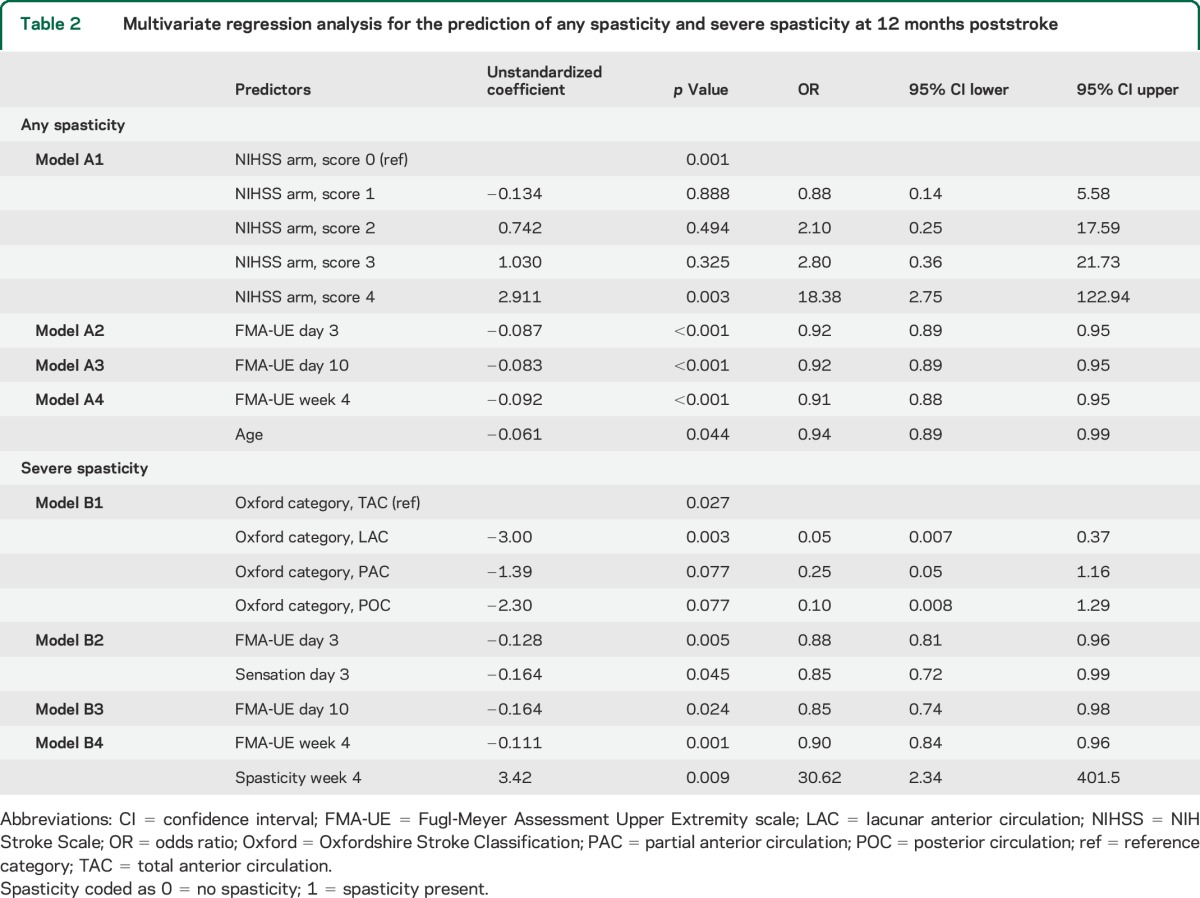
Multivariate regression analysis for the prediction of any spasticity and severe spasticity at 12 months poststroke

**Table 3:**
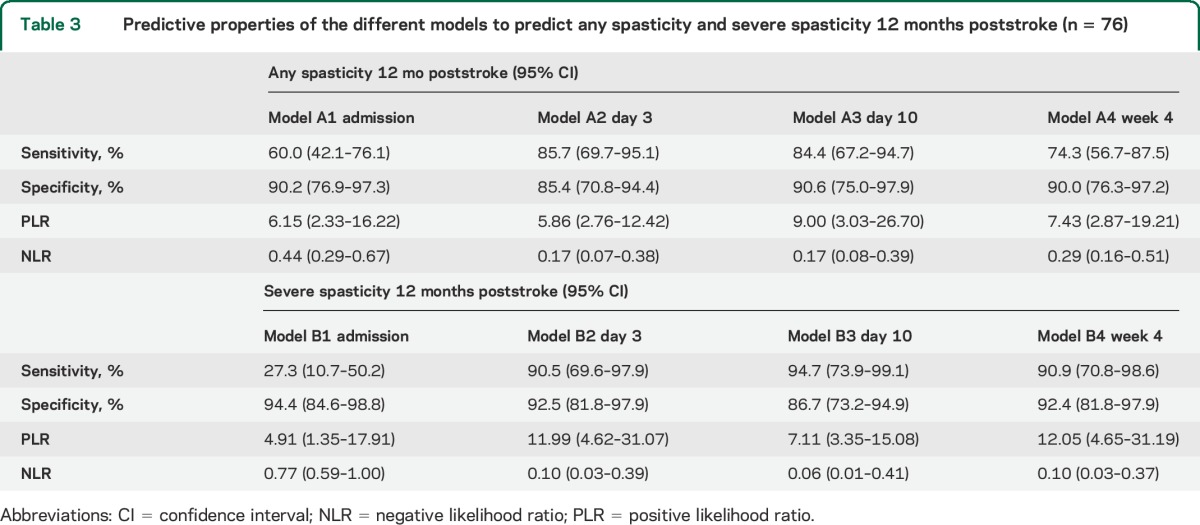
Predictive properties of the different models to predict any spasticity and severe spasticity 12 months poststroke (n = 76)

### Prediction of severe spasticity 12 months poststroke.

The results from multivariate logistic regression models B1–B4 are shown in table 2. Sensorimotor function (FMA-UE) was a significant predictor in all models, except B1. Lower FMA-UE scores (models B2–B4) were associated with an increased predicted probability for severe spasticity. In model B2 reduced sensation and in model B4 spasticity at 4 weeks poststroke were additional significant predictors. The sensitivity, specificity, PLR, and NLR of model B4 indicated that this model had the highest predictive value (table 3). The squared variables (FMA-UE^2^ and Sensation^2^) were not significant predictors in any of the models B2–B4, thus nonlinearity could not be shown.

## DISCUSSION

The present study demonstrated that any spasticity was best predicted with variables collected at day 10 poststroke and severe spasticity was best predicted with variables collected 4 weeks poststroke. Lower sensorimotor function score, as identified with the FMA-UE, consistently and significantly predicted both any spasticity and severe spasticity at 12 months poststroke. Age at stroke onset was a significant predictor 4 weeks poststroke, with higher age associated with reduced probability for spasticity. The presence of upper limb spasticity 4 weeks poststroke was a significant predictor for severe spasticity.

These findings support previous studies reporting paresis and reduced ADL function to be significant predictors for spasticity poststroke.^8,11,12^ However, none of the previous studies used the FMA-UE to assess sensorimotor function, and thus, a direct comparison cannot be made with those studies. Additionally, none of the former studies compared different prediction models for spasticity at 12 months poststroke based on the assessments at different time points relatively early after stroke onset, as was performed in the present study. Predicting upper limb spasticity with a relatively high accuracy based on early assessments may have high clinical relevance, as patients with an increased risk of developing spasticity-related impairments may be identified early and monitored more closely in order to implement appropriate interventions.

In the present study, patients with higher age were predicted to have reduced probability for spasticity. This finding may be in accordance with a previous study^22^ where more severe spasticity was found in younger patients 3 months poststroke, but not 18 months poststroke. Muscle force generation from tendon reflexes has been found to be slower and weaker with increasing age^27^ and if this also is the case for tonic reflexes associated with spasticity, spastic responses may be weaker in older patients.

In the present study, there was no association between the side of weakness and spasticity 1 year after stroke, as described previously.^12^ There may be several reasons for this discrepancy; for instance, there were methodologic differences between the studies, as the Tone Assessment Scale was used to assess spasticity, and spasticity and weakness were assessed in both arms and legs^12^ as opposed to only upper limb spasticity and sensorimotor function in the present study. An association between smoking and severe spasticity has been found previously,^12^ which could not be confirmed here. The authors discussed that their model, which included smoking, had an event per variable of 7, compared to the recommended minimum of 10.^12,28^

Model B1 showed that patients with lacunar stroke had a lower OR for severe spasticity than those with other stroke locations. Therefore, the Oxford categories were included in model B2 to check for stroke location as a possible predictive factor. No association between stroke location and spasticity was observed in model B2.

Presence of upper limb spasticity in the assessments during the first month was not a significant predictor for any spasticity at 12 months poststroke. Spasticity at 4 weeks poststroke was a significant predictor only for severe spasticity at 12 months. There may be both neurologic and muscular causes for this observation, as the tonic stretch reflexes may increase during the first 3 months, and intrinsic muscle changes may occur later.^29^ Therefore, spasticity may be an unstable impairment during the first months, before a more stable and manifest impairment is observed. A recent study based on the same study population supported this finding,^5^ as the authors found that patients changed both from no spasticity to any spasticity and vice versa during the first months poststroke.

Figure 2 shows the predicted probabilities in model A3 and the sensorimotor function at day 10, and indicates a fairly good fit of the model. A perfect agreement would have resulted in a straight, negative line, with FMA-UE = 0 equivalent to the highest probability (1.00), and FMA-UE = 66 equivalent to the lowest probability. Those scoring >40 points on the FMA-UE at day 10 had less than 20% probability for spasticity and those scoring <15 points had more than 80% probability for spasticity 12 months poststroke. There were relatively few patients scoring in the middle range (20–40 points) on the FMA-UE; therefore, the predictions of spasticity may be more uncertain in this range. The fit of model A3 was also confirmed by the relatively high sensitivity and specificity.

The clinical implications of the current study are mainly within 2 areas. First, the assessment of motor function at an early stage, either with the NIHSS at admission or with the FMA-UE, may give a good indication of the probability of a patient developing spasticity 12 months poststroke. At 3 days poststroke, the sensitivity and specificity of the prediction models were 85% and increased further at day 10. From a clinical perspective, the assessment of sensorimotor function and the early identification of patients at risk of developing spasticity and in particular severe spasticity may be important. Spasticity has been found to be associated with pain, reduced range of motion, and reduced motor function, which can have a negative impact on the functional ability of the patient.^1,5,13^ Patients at risk can be followed more closely, and spasticity may be treated both pharmacologically and non-pharmacologically.^30^ It is uncertain whether early treatment reduces spasticity in the long term. However, it has been well-established that such treatments have led to significant improvements in spasticity-related impairments, motor function, and quality of life among patients poststroke.^10,30–33^

The second clinical implication may come from the finding that the assessment of spasticity at day 3 or 10 could not predict spasticity after 1 year. Although the univariate logistic regression showed a significant association between spasticity at day 10 and spasticity 12 months poststroke, this association was lost in the multivariate regression analysis, as the FMA-UE was a much stronger predictor. Only after 4 weeks was the presence of spasticity a predictor, as patients with spasticity had a 30 times higher OR for severe spasticity 12 months poststroke than those without spasticity. Additionally, the chance of developing severe spasticity 1 year after stroke was very low if the assessment of spasticity at week 4 showed no spasticity. Consequently, there was a time point between 10 and 28 days poststroke at which spasticity became a predictor for severe spasticity, indicating that spasticity predominantly emerged and became manifest during this period. Therefore, the clinical value of assessing spasticity in patients much earlier than 4 weeks poststroke to predict long-term severe spasticity may be limited. However, the assessment of spasticity at this time may be important for other purposes. Both of these clinical implications indicate a need for regular and structured follow-ups for patients poststroke^34^ as spasticity and related impairments may develop over months.

The assessment of spasticity may be a limitation as the MAS is an ordinal, clinical assessment scale and not a metric measure of spasticity. However, it does not require any equipment, is easy to apply in different settings, is frequently used, and has relatively good intrarater reliability.^21^ In the present study, MAS was dichotomized for both any spasticity and severe spasticity, which may be claimed to be arbitrary and not necessarily coincide with important clinical divisions. The dichotomization for any spasticity has shown that patients with spasticity had poorer sensorimotor function, more pain, and reduced ROM than those without spasticity.^5^

In the current study, the summed score of the ordinal FMA-UE scale was used in the prediction models, which can be a limitation. However, the FMA-UE has been shown to have excellent psychometric properties, to be a valid indicator of motor recovery, and is widely used to indicate stroke severity.^35–37^ The unidimensional hierarchy of the FMA-UE has been demonstrated both in acute and chronic stroke^37,38^ and as nonlinearity of the FMA-UE could not be shown, we chose to use the FMA-UE in the analysis.

The patients in the present study may be regarded as fairly representative for patients with first stroke, with reduced arm function at day 3, living in a western European country, and receiving modern stroke care according to evidence-based practice. The patients in the present study may not be representative of the global population of persons poststroke.

## Supplementary Material

Data Supplement

Accompanying Comment

## GLOSSARY

ADL: activities of daily living
ARAT: Action Research Arm Test
CI: confidence interval
FMA-UE: Fugl-Meyer Assessment Upper Extremity Scale
MAS: modified Ashworth Scale
NIHSS: NIH Stroke Scale
NLR: negative likelihood ratio
PLR: positive likelihood ratio
ROM: range of motion
SALGOT: Stroke Arm Longitudinal Study at the University of Gothenburg
