# Supplementary material for: Early prediction of long-term upper limb spasticity after stroke: Part of the SALGOT study
Source: Neurology. 2015 Sep 8;85(10):873–80. doi: 10.1212/WNL.0000000000001908 (PMC4560058; doi:10.1212/WNL.0000000000001908)
Supplement: Data Supplement [file supp_WNL.0000000000001908_Tables.docx]

Table e-1. Univariate regression analysis for the prediction of any spasticity in the upper limbs 12 months poststroke.

|  | B | p-value | OR | 95% CI lower | 95% CI upper |
| --- | --- | --- | --- | --- | --- |
| Sex* | -0.181 | 0.701 | 0.834 | 0.331 | 2.104 |
| Age at onset of stroke* | 0.240 | 0.855 | 1.272 | 0.957 | 1.032 |
| Paretic arm, side* | 0.043 | 0.927 | 1.043 | 0.420 | 2.593 |
| Smoking* | -0.537 | 0.347 | 0.584 | 0.191 | 1.791 |
| Stroke type | -0.547 | 0.360 | 0.579 | 0.179 | 1.867 |
| Oxford cat* |  |  |  |  |  |
| TAC (ref) |  | 0.008 |  |  |  |
| LAC | -3.099 | 0.002 | 0.045 | 0.006 | 0.329 |
| PAC | -0.995 | 0.250 | 0.370 | 0.068 | 2.012 |
| POC | -1.253 | 0.274 | 0.286 | 0.030 | 2.692 |
| TOAST (n=98) |  |  |  |  |  |
| LAA (ref) |  | 0.216 |  |  |  |
| CE | 0.288 | 0.772 | 1.333 | 0.191 | 9.311 |
| SAO | 0.827 | 0.358 | 2.286 | 0.392 | 13.331 |
| OC | -0.668 | 0.472 | 0.513 | 0.083 | 3.158 |
| UND | -0.811 | 0.468 | 0.444 | 0.050 | 3.976 |
| NIHSS total | 0.224 | <0.001 | 1.251 | 1.119 | 1.399 |
| NIHSS arm,* score 0 (ref) |  | 0.001 |  |  |  |
| NIHSS arm, score 1 | -0.134 | 0.888 | 0.875 | 0.137 | 5.576 |
| NIHSS arm, score 2 | 0.742 | 0.494 | 2.100 | 0.251 | 17.594 |
| NIHSS arm, score 3 | 1.030 | 0.325 | 2.800 | 0.361 | 21.727 |
| NIHSS arm, score 4 | 2.911 | 0.003 | 18.375 | 2.746 | 122.944 |
| Sensation D3* | -0.252 | <0.001 | 0.777 | 0.692 | 0.872 |
| Sensation D10* | -0.229 | <0.001 | 0.795 | 0.708 | 0.893 |
| Sensation W4* | -0.311 | <0.001 | 0.733 | 0.624 | 0.860 |
| Joint pain D3 | -0.013 | 0.925 | 0.987 | 0.747 | 1.303 |
| Joint pain D10 | -0.127 | 0.412 | 0.880 | 0.649 | 1.194 |
| Joint pain W4* | -0.494 | 0.007 | 0.610 | 0.427 | 0.873 |
| ROM D3* | -0.276 | 0.142 | 0.759 | 0.525 | 1.096 |
| ROM D10* | -0.833 | 0.002 | 0.435 | 0.258 | 0.734 |
| ROM W4* | -1.142 | 0.002 | 0.319 | 0.152 | 0.668 |
| FMA-UE D3* | -0.087 | <0.001 | 0.916 | 0.887 | 0.947 |
| FMA-UE D10* | -0.083 | <0.001 | 0.920 | 0.890 | 0.951 |
| FMA-UE W4* | -0.079 | <0.001 | 0.924 | 0.896 | 0.953 |
| Spasticity D3* | 1.023 | 0.073 | 2.783 | 0.911 | 8.502 |
| Spasticity D10* | 2.017 | <0.001 | 7.515 | 2.684 | 21.042 |
| Spasticity W4* | 2.937 | <0.001 | 18.857 | 5.897 | 60.297 |

*Included in one or more multivariate analyses, also see figure 2. CI=confidence interval; D=day; W=week; Oxford cat= Oxfordshire Stroke Classification categories; TAC=Total Anterior Circulation; LAC=Lacunar; PAC=Partial Anterior Circulation; POC=Posterior Circulation; TOAST=Trial of Org 10172 in Acute Stroke Treatment; LAA=Large Artery Atherosclerosis; CE=Cardioembolism; SAO=Small Vessel Occlusion; OC=other determined cause; UND=undetermined cause; NIHSS=National Institute of Health Stroke Scale; ROM=Passive joint range of motion; FMA-UE= Fugl-Meyer Upper Extremity Assessment scale; Variable coding: sex: 0=male, 1=female; paretic arm: 1=right, 2=left; smoking: 0=no, 1=yes; stroke type: 0=Hemorrhagic, 1=ischemic; spasticity: 0=no spasticity, 1=spasticity present.

Table e-2. Univariate regression analysis for the prediction of severe spasticity in the upper limb 12 months poststroke.

|  | B | p-value | OR | 95% CI lower | 95% CI upper |
| --- | --- | --- | --- | --- | --- |
| Sex* | -1.430 | 0.020 | 0.239 | 0.072 | 0.801 |
| Age at onset of stroke* | -1.161 | 0.426 | 0.313 | 0.963 | 1.047 |
| Paretic arm, side | 0.411 | 0.430 | 1.509 | 0.544 | 4.184 |
| Smoking* | -0.298 | 0.641 | 0.742 | 0.212 | 2.604 |
| Stroke type | -0.768 | 0.210 | 0.464 | 0.139 | 1.542 |
| Oxfordshire categories* |  |  |  |  |  |
| TAC (ref) |  |  | 0.027 |  |  |
| LAC | -2.996 | 0.003 | 0.050 | 0.007 | 0.373 |
| PAC | -1.386 | 0.077 | 0.250 | 0.054 | 1.163 |
| POC | -2.303 | 0.077 | 0.100 | 0.008 | 1.288 |
| TOAST (n=98) |  |  |  |  |  |
| LAA (ref) |  | 0.637 |  |  |  |
| CE | -0.368 | 0.651 | 0.692 | 0.141 | 3.404 |
| SAO | -1.204 | 0.183 | 0.300 | 0.051 | 1.763 |
| OC | -0.693 | 0.505 | 0.500 | 0.065 | 3.845 |
| UND | -1.386 | 0.271 | 0.250 | 0.021 | 2.945 |
| NIHSS total | 0.179 | <0.001 | 1.196 | 1.085 | 1.318 |
| NIHSS arm,* score 0 (ref) |  | 0.01 |  |  |  |
| NIHSS arm, score 1 | -0.363 | 0.779 | 0.696 | 0.055 | 8.748 |
| NIHSS arm, score 2 | 1.569 | 0.223 | 4.800 | 0.385 | 59.895 |
| NIHSS arm, score 3 | 0.827 | 0.534 | 2.286 | 0.169 | 30.959 |
| NIHSS arm, score 4 | 2.321 | 0.041 | 10.182 | 1.102 | 94.104 |
| Sensation D3* | -0.292 | <0.001 | 0.747 | 0.658 | 0.847 |
| Sensation D10* | -0.230 | <0.001 | 0.795 | 0.709 | 0.891 |
| Sensation W4* | -0.272 | <0.001 | 0.762 | 0.673 | 0.862 |
| Joint pain D3 | 0.088 | 0.626 | 1.092 | 0.767 | 1.554 |
| Joint pain D10 | -0.188 | 0.229 | 0.828 | 0.610 | 1.126 |
| Joint pain W4* | -0.373 | 0.006 | 0.689 | 0.527 | 0.900 |
| ROM D3* | -0.293 | 0.108 | 0.746 | 0.522 | 1.066 |
| ROM D10* | -1.008 | <0.001 | 0.356 | 0.214 | 0.621 |
| ROM W4* | -1.012 | <0.001 | 0.363 | 0.207 | 0.637 |
| FMA-UE D3* | -0.144 | 0.002 | 0.866 | 0.791 | 0.948 |
| FMA-UE D10* | -0.164 | 0.024 | 0.849 | 0.737 | 0.978 |
| FMA-UE W4* | -0.113 | <0.001 | 0.893 | 0.847 | 0.943 |
| Spasticity D3* | 0.851 | 0.133 | 2.343 | 0.772 | 7.110 |
| Spasticity D10* | 2.062 | <0.001 | 7.862 | 2.473 | 25.001 |
| Spasticity W4* | 4.069 | <0.001 | 58.500 | 7.185 | 476.275 |

*Included in one or more multivariate analyses, also see figure 2. CI=confidence interval; D=day; W=week; Oxford cat= Oxfordshire Stroke Classification categories; TAC=Total Anterior Circulation; LAC=Lacunar; PAC=Partial Anterior Circulation; POC=Posterior Circulation; TOAST=Trial of Org 10172 in Acute Stroke Treatment; LAA=Large Artery Atherosclerosis; CE=Cardioembolism; SAO=Small Vessel Occlusion; OC=other determined cause; UND=undetermined cause; NIHSS=National Institute of Health Stroke Scale; ROM=Passive joint range of motion; FMA-UE= Fugl-Meyer Upper Extremity Assessment scale; Variable coding: sex: 0=male, 1=female; paretic arm: 1=right, 2=left; smoking: 0=no, 1=yes; stroke type: 0=Hemorrhagic, 1=ischemic; spasticity: 0=no spasticity, 1=spasticity present.
