# Supplementary material for: Early prediction of long-term upper limb spasticity after stroke: Part of the SALGOT study
Source: Neurology. 2015 Sep 8;85(10):873–80. doi: 10.1212/WNL.0000000000001908 (PMC4560058; doi:10.1212/WNL.0000000000001908)
Supplement: Accompanying Comment [file supp_85_10_873_v2_index.html]

Early prediction of long-term upper limb spasticity after stroke — Accompanying Comment 

# Early prediction of long-term upper limb spasticity after stroke

## Accompanying Comment

**Neurology® data supplements are not copyedited before publication. Published editorials and translations have been copyedited.  
 © 2015 American Academy of Neurology.  
  
 Files in this Data Supplement:**

- Accompanying Comment - PDF
