# Supplementary material for: Early prediction of long-term upper limb spasticity after stroke: Part of the SALGOT study
Source: Neurology. 2015 Sep 8;85(10):873–80. doi: 10.1212/WNL.0000000000001908 (PMC4560058; doi:10.1212/WNL.0000000000001908)
Supplement: Accompanying Comment [file supp_WNL.0000000000001908_WNL.0000000000001917.pdf]

Comment:
How and why to predict spasticity after stroke?

Although many stroke patients present with spasticity, this impairment remains a riddle for physicians. Why, when, and how does a patient develop spasticity, whereas another patient with a similar cerebral lesion does not? Moreover, the evolution of spasticity among these chronic patients and its relation to functional activity are not straightforward. Thus, the assessment and treatment of spasticity remain a challenge in neurorehabilitation.

Opheim et al.[1] identify the early predictors of spasticity among stroke patients: age, sex, and neurologic impairments assessed with the Fugl-Meyer scale. Assessing the patient 10 days and 4 weeks after stroke allows the prediction, respectively, of the presence of spasticity and its severity at 1 year poststroke. Interestingly, stroke severity assessed by the NIH Stroke Scale at admission was not a predictor. This emphasizes the importance of assessing patients regularly and accurately during rehabilitation. Ideally, this assessment should not focus only on neurologic impairments. Following the WHO International Classification of Functioning, Disability, and Health (www.who.int/classifications/icf), the activities that the patient performs in his or her environment and his or her social participation should also be assessed.

This study also underlines the usefulness of the Fugl-Meyer scale. However, whereas the authors used the original ordinal scale, they submitted the results to complex statistical methods. The Fugl-Meyer scale, as many other scales used in neurorehabilitation,[2] has been transformed to a linear scale through Rasch analysis. In clinical practice and future research, it would be preferable to use these improved versions to optimize the quality of assessment and to gather continuous data suitable to powerful parametric statistics.[3,4]

Early identification of patients at risk of developing spasticity should improve the quality of care. They should be regularly assessed and would benefit from early treatment to avoid long-term complications (e.g., contractures), especially for the most impaired patients or those with reduced access to specialists.

1. Opheim A, Danielsson A, Alt Murphy M, Persson HC, Sunnerhagen KS. Early prediction of long-term upper limb spasticity after stroke: part of the SALGOT study. Neurology 2015;85:xxx–xxx.
2. Belvedere SL, Morton NA. Application of Rasch analysis in health care is increasing and is applied for variable reasons in mobility instruments. J Clin Epidemiol 2010;63:1–11.
3. Grimby G, Tennant A, Tesio L. The use of raw scores from ordinal scales: time to end malpractice? J Rehabil Med 2012;44:97–98.
4. Smith AG, Burns TM. Clinical measurement tools in therapeutic trials: time to make a Rasch decision? Neurology 2014;83:2104–2105.

Thierry M. Lejeune, MD, PhD

Gaëtan Stoquart, MD, PhD

From the Physical Medicine and Rehabilitation Department, Cliniques Universitaires Saint-Luc, Université Catholique de Louvain, Brussels, Belgium.

Study funding: No targeted funding reported.

Disclosure: The authors report no disclosures relevant to the manuscript. Go to Neurology.org for full disclosures.

© 2015 American Academy of Neurology. Unauthorized reproduction of this article is prohibited.
